# Paediatric Enterobacteriaceae infections in hospitalised children in Durban, KwaZulu-Natal

**DOI:** 10.4102/sajid.v36i1.279

**Published:** 2021-07-30

**Authors:** Harshna Krishinchand, Kimesh Naidoo, Prasha Mahabeer, Moherndran Archary

**Affiliations:** 1 Department of Paediatrics and Child Health, School of Clinical Medicine, College of Health Sciences, University of KwaZulu-Natal, Durban, South Africa; 2 Department of Paediatrics, King Edward VIII Hospital, Durban, South Africa; 3 Department of Microbiology, King Edward VIII Hospital, Durban, South Africa; 4 Department of Medical Microbiology, School of Laboratory Medicine and Medical Sciences, College of Health Sciences, University of KwaZulu-Natal, Durban, South Africa; 5 Department of Paediatrics and Child Health, King Edward VIII Hospital, Durban, South Africa

**Keywords:** paediatric, Enterobacteriaceae infections, HIV, malnutrition, hospitalisation

## Abstract

**Background:**

Community-acquired Gram-negative Enterobacteriaceae infections in malnourished and HIV-infected hospitalised children are not well documented and are of concern because of increasing antibiotic resistance and limited available treatment options. This study describes the clinical characteristics and outcomes of hospitalised children with positive Enterobacteriaceae cultures.

**Method:**

A retrospective chart review of children with Gram-negative Enterobacteriaceae infections was performed in King Edward VIII Hospital, a referral hospital in Durban, KwaZulu-Natal. Standard descriptive and analytical statistics, including regression analysis, were performed to determine the clinical characteristics associated with Enterobacteriaceae infections in children hospitalised in the study period.

**Results:**

Of all hospitalised children in the study period, 207 (3.5%) had positive cultures for Enterobacteriaceae isolates, with *Escherichia coli* 109 (44.5%) and *Klebsiella* spp. 59 (24.1%) making up most of the infections. Urine (126; 58%) followed by stool (34; 14.8%) and blood (35; 14.0%) were the commonest samples that yielded positive cultures. Diarrhoeal hospitalisations especially posed a higher risk for Enterobacteriaceae infections. Severe acutely malnourished and HIV-infected children were at higher risk. These comorbidities were independently associated with an increased risk of Enterobacteriaceae infection. Prolonged hospitalisation and increased risk of death were also associated with Enterobacteriaceae infection.

**Conclusion:**

Enterobacteriaceae infections were common in hospitalised children and posed an increased risk, especially in malnourished and HIV-infected children. Further studies investigating the relationships between diarrhoea, urinary tract infections and Enterobacteriaceae infections are needed.

## Introduction

Sub-Saharan Africa continues to have high under-five mortality rates fuelled by the burdens of malnutrition and multiple childhood infections.^1,2,3^ Gram-negative Enterobacteriaceae infections are of particular concern because of an increasing incidence and the emergence of broad-spectrum antibiotic resistance, limiting the available treatment options.^4,5^ The extent of Gram-negative Enterobacteriaceae infections has been well documented in adults, but this is not the case in children, especially in populations with high HIV and malnutrition rates.^6,7^

Severely malnourished children have a threefold higher case fatality than children with normal nutritional status.^8^ Although the current standard management guidelines for children hospitalised with severe acute malnutrition (SAM) recommend empiric antibiotics (ampicillin and gentamicin or ceftriaxone for severe illness), these antibiotics may be less effective in treating Enterobacteriaceae infections.^9,10^ Understanding the incidence and antimicrobial resistance patterns of Gram-negative isolates from malnourished children will better inform antibiotic usage in malnourished children and will influence the choice of antibiotics in this vulnerable population.

Invasive bacterial enteric pathogens, including *Shigella, Campylobacter* and Enteroaggregative-adherent *E. coli* (EAEC), have been associated with gut inflammation and barrier disruption triggering chronic diarrhoea that can lead to malabsorption and malnutrition.^11^

Multiple factors such as organ or stem cell transplant, intensive care unit admission, prolonged hospitalisation, surgery and care in long-term care facilities have been highlighted as risk factors for infection with drug-resistant Enterobacteriaceae in adults.^12^

In a review summarising six studies of carbapenem-resistant Enterobacteriaceae infections, 64 isolates were cultured from 63 children. The median age of infected children was 1 year (0–17 years), males (67%) were affected more than females. Many children (53%) were hospitalised to an intencive care unit (ICU) and probably had hospital-acquired infections. A total of 87% had been hospitalised for more than 48 h. Comorbidities identified included pulmonary disease, prematurity, leukaemia or solid tumours, cardiac disease, necrotising enterocolitis, solid organ or stem cell receipt.^13^

Extended-spectrum β-lactamase (ESBL) producing Enterobacteriaceae have emerged as a major cause of healthcare-associated infections with significant morbidity and mortality in children.^12,13^.

In populations with high HIV endemicity, children are hospitalised with poor nutritional status and opportunistic infections, including tuberculosis. The relationship to Enterobacteriaceae infection has not been completely described and explained.

There are limited therapeutic options available for multidrug resistant Gram-negative bacterial infections in children.^7^ Ideal treatment regimens are hampered by a scarcity of randomised trials assessing the efficacy of various drug options in children with community-acquired Enterobacteriaceae infections.^14,15^ Morbidity and mortality associated with these infections are high. Reports on epidemiology and risk factors for infection in children are limited.^7,12,13,15^

This study aimed to describe the clinical characteristics of children who were hospitalised at King Edward VIII Hospital (KEH), Durban, from whom an Enterobacteriaceae was cultured.

## Methodology

A retrospective, descriptive, observational study of all children hospitalised at KEH, who cultured an Enterobacteriaceae from any site, was conducted from the period January 2016 to 31 December 2017. All hospitalised children are admitted to an acute admission ward under the care of a specialist paediatrician. Decisions on specimen collection for specific microbiological cultures were made at the discretion of the treating clinician.

The National Health Laboratory Service (NHLS) was used to access all positive Enterobacteriaceae cultures, from any site, in children between the ages of > 28 days and < 13 years for the study period. This study excluded neonates (who have a higher Gram-negative burden of infections) and reflects a general hospitalised paediatric population with an expected lower burden of such infections.

The patient files of all children who had positive Enterobacteriaceae cultures were sourced from the KEH archives, and the following data were extracted: age, gender, final clinical diagnosis, HIV status, nutritional status, clinical outcome, mortality and length of stay (LOS). Anthropometric measurements from the patient files were used to classify nutritional status using World Health Organisation (WHO) growth standards. Patients were classified: no malnutrition (NAM), moderate acute malnutrition (MAM), severe acute malnutrition (SAM) (with or without oedema) and overweight for age (OWFA).^16^

The HIV status of infants was determined from the last verified HIV test carried out within the health system and results were also verified on the NHLS information system. They were categorised as: HIV-infected, HIV-uninfected, HIV-exposed uninfected and HIV-exposed unknown. For the exposed group without confirmation of HIV results, these patients were categorised as unknown (no confirmation of HIV results). Patients infected with HIV were further categorised as receiving antiretroviral treatment (ART) or not. Patients with an unknown HIV result (107/5946; 1.8%) were excluded from the non-descriptive analysis.

Information of the sites of culture, types of organisms and antibiotic sensitivity were extracted and verified from the NHLS information system. All data were extracted into an excel spreadsheet.

Data from the study cohort was compared with routinely collected data on all children hospitalised to the paediatric medical wards at KEH during the study period.

### Data analysis

Descriptive and inferential statistical analysis methods were used for understanding and deriving meaningful conclusions from the collected data. Summary measures such as the minimum, maximum, quartiles, interquartile range, mean, standard deviation and coefficient of variation were calculated for the descriptive statistics. The categorical variables were described as counts and percentage frequencies. To assess the mean difference of numerical variables across at least three levels of a categorical variable, the ANOVA test for normally distributed measurements or the Kruskal–Wallis test for the non-normally distributed measurements were used. To test the association between categorical variables, the Chi-square test or Fisher’s exact test (small frequencies) were used. All the inferential statistical analysis tests were conducted at 5% levels of significance.

### Ethical considerations

Ethical approval was obtained from the University of KwaZulu-Natal (UKZN) Biomedical Research Ethics Committee (BREC Ref No: BE 125/19) and gatekeeper permission from the National Health Laboratory Service (NHLS) and King Edward VIII Hospital (KEH).

## Results

A total of 5946 children above 1 month were hospitalised to King Edward Hospital Paediatric Department during the study period 01 January 2016 and 31 December 2017. In this period, 207 of these hospitalised children (3.5%) had positive Enterobacteriaceae cultures, of which 59.4% were community-acquired (defined as positive cultures less than 48 h after admission) and 40.6% were hospital-acquired defined as positive cultures more than 48 h after admission.

**FIGURE 1:**
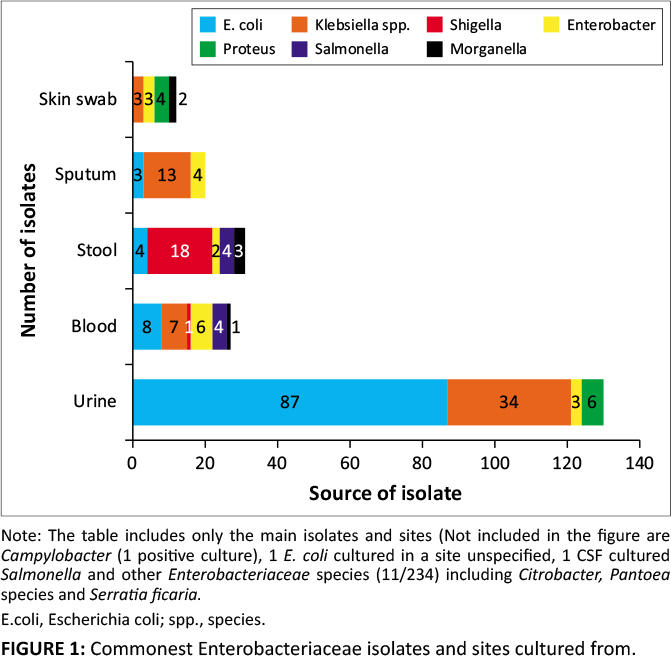
Commonest Enterobacteriaceae isolates and sites cultured from.

Figure 1 illustrates the most common organisms and the sites from which they were cultured. *Escherichia coli* (109; 44.5%) and *Klebsiella* spp. (59; 24.1%) were the most commonly isolated organisms. The positive cultures were obtained from urine (135/234; 57.7%), stool (33/234; 14.1%), blood (33/234; 14.1%), sputum (21/234; 9%), skin swabs (10/234; 4.3%), CSF (1; 0.4%) and one site (1; 0.4%), which was unknown *E. coli* was isolated predominately from urine and *Klebsiella* spp. from sputum. Extended-spectrum β-lactamase producing organisms were found in 51 patients, 33 (65.0%) of whom were malnourished and 18 (35.0%) HIV-infected. Only two of the 207 patients cultured extensively drug-resistant organisms (defined as non-susceptibility to at least one agent in three or more antimicrobial categories). These were both *Klebsiella* spp. cultured from urine. Of the 59 isolates that were cultured ESBL producers, these were: *Klebsiella* spp. (27/59; 45.8%), *E. coli* (26/59; 44.1%) and the remaining 10% were *Shigella* (2/59), both from stool samples and *Morganella* (1/59) cultured from sputum.

### Clinical characteristics of patients with Enterobacteriaceae infections

Five patients in the study group were re-hospitalised within 8 weeks of discharge, and 13 (6.3%) patients in the study group were transferred from other healthcare facilities. In some patients (33; 15.9%), Enterobacteriaceae were cultured on more than one occasion during the same hospitalisation period. Seventeen samples were repeat samples.

In this public sector hospital, all paediatric hospitalisations are categorised into three main diagnostic groups: respiratory illness, diarrhoeal disease and ‘other’ (includes meningitis, urinary tract infection [UTI], septicaemia and others). Table 1 highlights that Enterobacteriaceae infections were significantly more common in children hospitalised with the diarrhoeal diagnosis. Table 1 compares the clinical characteristics of patients admitted with and without Enterobacteriaceae infections.

**TABLE 1:** Age, gender, main diagnosis and Enterobacteriaceae infections.

Group	No Enterobacteriaceae infection		Enterobacteriaceae infected		*p*
	*N* = 5739	%	*N* = 207	%	
**Gender**	-		-		0.078
Female	2364	41.2	98	47.3	-
Male	3373	58.8	109	52.7	-
Missing	2	0.0	0	0	-
**Age**	-		-		0.009
28 days – < 1 year	2014	35.1	59	28.5	0.120
1 year – < 5 years	2538	44.2	114	55.1	0.008
5 years – < 13 years	1187	20.7	34	16.4	0.161
**Main diagnosis**	-		-		< 0.001
Respiratory illness	1780	31.0	24	11.6	< 0.001
Diarrhoeal disease	863	15.0	93	44.9	< 0.001
Other	3095	53.9	90	43.5	0.004
Missing	1	0.0	0	0	-

The *p*-values are based on non-missing cases only.

### Nutritional status

Children hospitalised with SAM (with or without oedema) showed a significantly increased risk of Enterobacteriaceae infection (Table 2).

**TABLE 2:** Nutritional status and Enterobacteriacae infections.

Nutritional status	No Enterobacteriaceae infection		Enterobacteriaceae infection		*p* < 0.001
	*N*	%	*N*	%	
Normal	4672	82.4	119	57.5	< 0.001
OWFA	244	4.3	6	2.9	0.480
UWFA	400	7.1	22	10.6	0.110
SAM with oedema	279	4.9	32	15.5	< 0.001
SAM without oedema	75	1.3	28	13.5	< 0.001
Missing	1	0.0	0	0	-

OWFA, overweight for age; UWFA, underweight for age; SAM, severe acute malnutrition.

The *p*-values are based on non-missing cases only.

**FIGURE 2:**
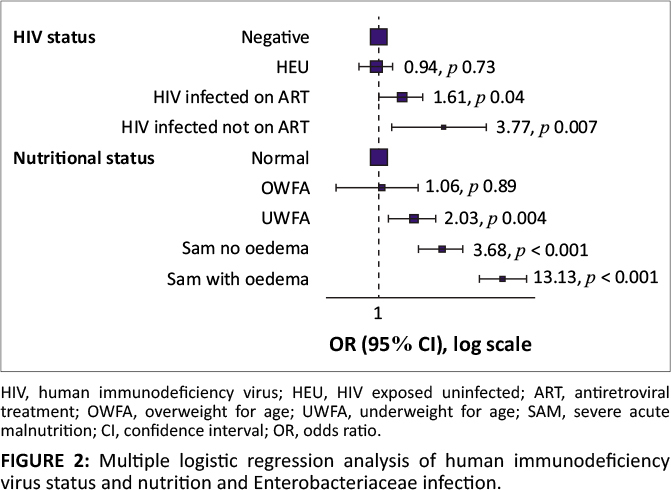
Multiple logistic regression analysis of human immunodeficiency virus status and nutrition and Enterobacteriaceae infection.

Children classified as SAM with oedema were the group most susceptible to Enterobacteriaceae infection, and this is shown by the logistic regression analysis in Figure 2.

### Human immunodeficiency virus exposure

Of the 207 Enterobacteriaceae culture-positive patients, 39 (17.1%) were HIV-infected. Of these, 33 were on lifelong antiretroviral treatment (ART).

Of the HIV-infected children, a greater number of those who were not on ART had an Enterobactericiae infection(6/35; 17.1%) when compared with HIV-infected children on ART (33/413; 8%) (*p* < 0.001). The duration of ART, CD4 count and viral load data were not available for patients in either of these groups. Table 3 indicates the HIV status of children with and without Enterobacteriaceae infection.

**TABLE 3:** Human immunodeficiency virus status and Enterobacteriacae infections.

HIV status	No Enterobacteriaceae infection		Enterobacteriaceae infected		*p* < 0.001
	*N*	%	*N*	%	
Negative	3607	63.6	112	54.1	0.010
HIV-exposed uninfected	1599	28.2	55	26.6	0.637
Infected on cART	380	6.7	33	15.9	< 0.001
Infected not on cART	29	0.5	6	2.9	0.004

HIV, human immunodeficiency virus; cART, combination antiretroviral treatment.

The *p*-values are based on non-missing cases only.

### Comparison of nutritional status and human immunodeficiency virus infection risk to Enterobacteriaceae infections

Figure 2 illustrates that HIV infection and malnutrition were independently associated with Enterobacteriaceae infection. Severe acute malnutrition with oedema was significantly associated with Enterobacteriaceae infections. Human immunodeficiency virus infection also posed a higher risk, less if the HIV-infected child was on ART.

### Duration of stay

The mean LOS for all hospitalisations for the study period was 5.5 days, whereas 99 (47%) children infected with Enterobacteriaceae stayed for longer than 7 days and more than half of these children (54; 26.1%) were hospitalised for at least 2 weeks.

### Mortality

Mortality was more than twice higher in children with Enterobacteriaceae infection (10; 4.8%) compared with those who did not have an Enterobacteriaceae infection (113; 2.0%) *p* = 0.005. Of the children who died because of Enterobacteriaceae infection, half had a positive blood culture (*n* = 5), with the rest having non-invasive isolates (two sputum isolates, one urine isolate, one skin swab isolate, and one death had an unspecified isolate).

## Discussion

This study evaluated the clinical characteristics of hospitalised children with a positive Enterobacteriaceae culture. Human immunodeficiency virus and malnutrition were significant risk factors for Enterobacteriaceae infection.

The association of HIV infection and increased risk for Enterobacteriaceae infection in children is not well understood, especially in the context of improved ART access.^1,2^ Human immunodeficiency virus-exposed infants may have lower body weight, an ill mother and thus may be at risk for poor growth trajectories and increased infections. Higher infection rates with Enteropathogenic *E. coli* (EPEC), Cryptosporidium and increased gut colonisation with ESBL Enterobacteriaceae have been found in HIV-infected children.^17,18,19,20^ In our study, the incidence of Enterobacteriaceae infection was higher in children who were yet to be initiated on ART compared with those already on lifelong ART. Being ART naïve is probably associated with a compromised immune system. The independent association of HIV and malnutrition with Enterobacteriaceae infection in this study highlights the contributing role of these factors to infection risk.

In this cohort, children with SAM with or without oedema were more susceptible to Enterobacteriaceae infection. Several studies have described that patients with malnutrition are more vulnerable to Enterobacteriaceae infections.^21,22,23,24^ Environmental enteric dysfunction (EED), villous blunting, reduction in mucus-secreting goblet cells, inflammation, reduced microbial diversity and increased potent pathogenic Enterobacteriaceae in children with malnutrition have been postulated as potential causes and warrant further study.^25,26,27,28,29^

In keeping with other studies, the authors found that diarrhoeal disease was strongly associated with Enterobacteriaceae infection.^24,30,31^ These findings suggest that Enterobacteriaceae infection, malnutritions and diarrhoea are possibly intertwined.

The incidence of Enterobacteriaceae infection in this study (3.5%) is similar to reports from other studies.^15,32,33^ The higher incidence of Enterobacteriaceae infections in children under five has also been previously reported, and the role of perineal hygiene and toilet training are considered possible contributory factors.^34^

Positive cultures for Enterobacteriaceae infections were predominantly from urinary samples. Several studies have demonstrated that Enterobacteriaceae organisms are the dominant Gram-negative organism causing UTIs in children.^35,36,37^ Urinary tract infections in children often present with non-urinary tract symptoms and are frequently diagnosed during investigations for sepsis, diarrhoea or malnutrition.^38^ The association between UTIs and malnutrition has been documented and the authors of this study confirms this association.

Enterobacteriaceae are the leading group of Gram-negative organisms causing UTIs in children, which has implications for the empiric choice of antibiotics.^39^ The South African Standard Treatment Guidelines recommend using amoxicillin/clavulanic acid to treat UTIs and ampicillin and gentamicin to treat children with complicated SAM or oral amoxicillin for uncomplicated SAM.^10^ The emergence of increasing antibiotic drug resistance, especially in hospitalised children with malnutrition, is of concern. The guidelines may need to be revised.

Increasing antibiotic resistance amongst urinary tract isolates against ampicillin, amoxicillin and cotrimoxazole has been reported. Most isolates are still sensitive to third-generation cephalosporins, quinolones, macrolides and aminoglycosides.^40^ Careful monitoring of antimicrobial resistance in high-risk sub-populations, such as severely malnourished children, is warranted.

Of the 207 Enterobacteriaceae cultured, 51 (24.6%) produced extended-spectrum beta-lactamases (ESBL) in mostly community-acquired infections.

This is in keeping with other studies, where an incidence of 10% – 35% was described.^7,17,33^ A recent study demonstrated the correlation between the routine use of amoxicillin in children with uncomplicated SAM and increased ESBL production.^41^ Amoxicillin use is recommended as one of the WHO ‘10 steps’ in managing malnutrition.^42^ Children exposed to amoxicillin showed an increased ESBL production amongst isolates and a higher risk of transmission to siblings.^41^ Further research on the use of broad-spectrum antibiotics amongst malnourished children is required.^43,44,45^

### Limitations

This was a retrospective study and data were obtained from patient records, where there was no standardisation of anthropometric measurements, blood and urine sampling techniques and clinical reviews. Information on ART duration was not available, CD4 count and HIV viral load results were also not available for analysis. There were missing data, including confirmatory HIV PCR results. In this study, comparisons of infections from community-acquired and hospital-acquired and from invasive (blood, CSF) and non-invasive (urine, stool) sources could not be determined. In addition, the susceptibility of empiric choices in antibiotics could not be confirmed.

## Conclusion

This study was conducted in an area with a high prevalence of HIV infection. Enterobacteriaceae infections are relatively common in hospitalised children, particularly in severely malnourished children with oedema and HIV-infected children not on ART. A high index of suspicion is necessary when caring for hospitalised children with these diagnoses. Revision of current antibiotic recommendations for hospitalised children with malnutrition may be necessary if current guidelines result in treatment failure related to antimicrobial resistance. Strategies to prevent malnutrition and HIV infection in children should be prioritised to reduce the risk of Enterobacteriaceae infections, mortality and prolonged hospital stay.
